# Catastrophic outcome of patients with a rebound after Natalizumab treatment discontinuation

**DOI:** 10.1002/brb3.671

**Published:** 2017-03-14

**Authors:** Inés González‐Suarez, Luis Rodríguez de Antonio, Aida Orviz, Sara Moreno‐García, María D. Valle‐Arcos, Jordi A. Matias‐Guiu, Cristina Valencia, Manuela Jorquera Moya, Celia Oreja‐Guevara

**Affiliations:** ^1^ Neurology DepartmentMultiple Sclerosis CenterIdiSSCHospital Clinico San CarlosMadridSpain; ^2^ Neurology DepartmentHospital Universitario de FuenlabradaMadridSpain; ^3^ Demyelinating Disease UnitNeurology DepartmentHospital Universitario 12 de OctubreMadridSpain; ^4^ Radiology DepartmentIdiSSCHospital Clinico San CarlosMadridSpain

**Keywords:** immune reconstitution inflammatory, Natalizumab withdrawal, rebound, syndrome

## Abstract

**Introduction:**

Natalizumab (NTZ) is an effective drug for the treatment of relapsing‐remitting multiple sclerosis. In some patients discontinuation is mandatory due to the risk of progressive multifocal leukoencephalopathy. However, severe clinical and radiological worsening has been described after drug cessation. Our aim was to describe the clinical and radiological features of the rebound phenomenon.

**Material and Methods:**

Patients switched from NTZ to Fingolimod (FTY) who had presented a rebound after discontinuation were selected. Clinical and magnetic resonance imaging (MRI) data were collected.

**Results:**

Four JC virus positive patients were included. The mean disease duration was 9.5 years (*SD*: 4.12) with a mean time of 3.1 years on NTZ. All patients started FTY within 3–4 months. Neurological deterioration started in a mean time of 3.5 months (*SD*: 2.08) with multifocal involvement: 75% motor disturbances, 50% cognitive impairment, 25% seizures. The average worsening in Expanded Disability Status Scale [EDSS] was of 3.25 points (*SD*: 2.33). The MRI showed a very large increase in T2 and gadolinium‐enhanced lesions (mean: 23.67, *SD*: 18.58). All patients received 5 days of IV methylprednisolone, one patient required plasma exchange. All the patients presented neurological deterioration with an EDSS worsening of 1.13 points (*SD*: 0.48). After the rebound three patients continued treatment with FTY, only one patient restarted NTZ.

**Conclusion:**

Discontinuation of NTZ treatment may trigger a severe rebound with marked clinical and radiological worsening. A very careful evaluation of benefit‐risk should be considered before NTZ withdrawal, and a close monitoring and a short washout period is recommended after drug withdrawal.

## 1 Introduction

Natalizumab (NTZ) is a human monoclonal antibody against α4 integrin, its mechanism of action blocks this protein and inhibits the entry of inflammatory cells into the brain (Engelhardt & Kappos, 2008). It is considered a very effective drug as it reduces the relapse rate and the disease activity on MRI dramatically and also moderately reduces the progression of disability; it is the treatment of choice for patients with aggressive multiple sclerosis (MS) or poor response to the first‐line therapy (Goodman et al., 2009; Miller et al., 2003; Polman et al., 2006). However, its use has been limited due to the risk of opportunistic infections, highlighting the progressive multifocal leukoencephalopathy (PML) secondary to JC virus reactivation. PML is a demyelinating disease of the CNS with frequent fatal consequences and with an incidence of approximately 1:1,000 in the treated patients (Yousry et al., 2006). The risk increases with longer treatment duration, the presence of anti‐JCV antibodies and the prior use of immunosuppression (Fernandez et al., 2015; Yousry et al., 2006). 638 PML cases have been reported with an incidence of 4.15/1,000 in treated patients (Biogen Idec data, March 2016, website http://www.biogenidec-international.com/tysabri.aspx?). Therefore, in some cases, the risk is not acceptable and the discontinuation of NTZ is mandatory; the European Medicines Agency and US Food and Drug Administration have established a risk management plan, and a re‐consent of all patients treated for longer than 2 years is recommended (2016; Fernandez et al., 2015).

Frequently, after NTZ cessation the activity of the disease returns to pretreatment levels (Fox et al., 2014; O'Connor et al., 2011); however, some cases with severe clinical and radiological worsening have been described (Beume et al., 2015; Daelman et al., 2012; Gueguen et al., 2014; Jander et al., 2012; Kerbrat et al., 2011; Lenhard et al., 2010; Miravalle et al., 2011; Papeix et al., 2011; Rigau et al., 2012; Rinaldi et al., 2012; Salhofer‐Polanyi et al., 2014; Sorensen et al., 2014; Vellinga et al., 2008; West & Cree, 2010) probably related to immune reconstitution inflammatory syndrome (IRIS; Lenhard et al., 2010; Miravalle et al., 2011).

## 2 Patients and Methods

From a cohort of NTZ‐treated patients from three hospitals in Madrid, Spain, between 2012–2013; those switched to Fingolimod (FTY) who had presented a rebound after discontinuation were reported. Clinical and magnetic resonance imaging (MRI) data were collected.

### 2.1 Case report 1

**Figure 1:**
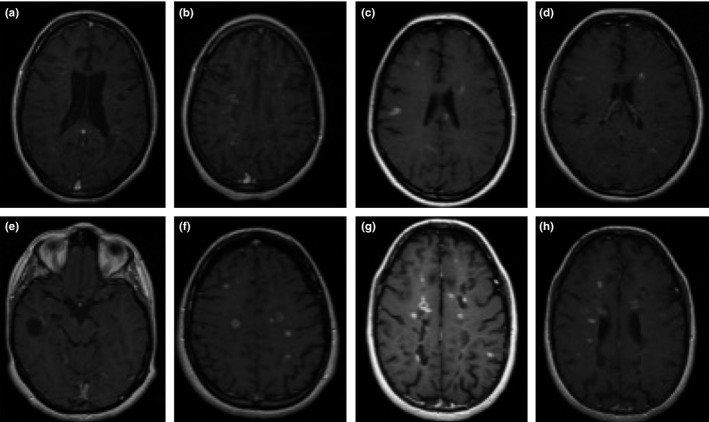
Axial T1 weighted magnetic resonance imaging (MRI) images after gadolinium (Gd) administration. The images demonstrated a high inflammation activity with elevated load of Gd‐enhancing lesions. (a) and (b) Patient 1; (c) and (d) patient 2; (e) and (f) patient 3; (g) and (h) patient 4

A 29‐year‐old male was diagnosed with relapsing‐remitting MS (RRMS) in 1999. He was treated with interferon beta until 2010. He started NTZ due to two relapses in previous year (no gadolinium [Gd] enhanced lesions in MRI). In February 2012 NTZ was discontinued due to the presence of 2 risk factors for PML (26 NTZ infusions and anti‐JCV+). At this time, he had mild left hemiparesis and needed a cane for ambulation (Expanded Disability Status Scale [EDSS]: 6) but he was fully independent. In June 2012 FTY was started after a washout period of 3 months without adverse effects. One day later the patient was admitted to the emergency room due to drowsiness, disorientation, inattention, bradypsychia, and behavioral changes. 15 days' prior the patient had had a worsening in his left hemiparesis. On admission, neurological examination revealed a moderate encephalopathy with disorientation, severe inattention and bradyspsychia; Language was less fluent without dysphasia, he comprehended simple commands but not complex ones. He presented a worsening of his left hemiparesis with strength of 4/5 in left upper limb and 3/5 in left lower limb with generalized hyperreflexia and severe dysmetria, the patient was unable to stand up with aid (EDSS: 7). The MRI showed confluent bilateral white matter T2 hyperintensities with enhancement of multiple lesions (Figure 1a,b), also a Vth and VIIth nerve enhancement bilaterally. This enhancement was prolonged further. Spinal fluid examination was normal with absence of JCV by PCR. High‐dose methylprednisolone (MTP) for 5 days was started with progressive improvement. Fingolimod was restarted without new relapses but with a marked deterioration in comparison to his prior situation (EDSS:7).

### 2.2 Case report 2

A 28‐year‐old female was diagnosed with RRMS in 2006. She was treated with interferon, Glatiramer Acetate and since September 2009 (a severe motor relapse the previous year) with NTZ. She presented no evidence of clinical and radiological activity in 2 years. In September 2012 NTZ was stopped due to a positive serology for VJC (EDSS: 1). Within the first month after the discontinuation the patient showed progressive worsening with paresthesia, instability, dizziness, and occasional diplopia. She was treated with several IV steroid pulses (MTP 13gr in total); in January 2013 FTY was started without response (EDSS: 6.5). An MRI revealed multiple T2 hyperintensities, of which at least 11 showed enhancement after Gd administration (Figure 1c,d). Due to the progressive worsening despite the treatment, the patient was admitted for plasma exchange (PLEX) with progressive clinical and radiological improvement. She was discharged and FTY was continued with a partial recovery (EDSS: 2.5).

### 2.3 Case report 3

A 38‐year‐old female diagnosed with RRMS in 1999. She was treated with Interferon B 1b with unsatisfactory control (two relapses in 2007), so in January 2008 the patient switched to NTZ. In September 2011, she presented an optic neuritis and was treated with high doses of MTP. In 2012 due to a high risk for PML a change to FTY was proposed. She received the last dose of NTZ in June 2012 (EDSS: 2.0). After a 3‐month washout period she started FTY with no secondary effect. In the last week of December 2012, she presented two partial seizures separated by 6 days and she was treated with levetiracetam. In the MRI of January 2013 multiple new lesions were observed in T2, 15 of them presented enhancement post Gd administration (Figure 1e,f). There were no signs of PML on MRI. Two weeks later she presented a marked worsening with severe paraparesis with moderate dysmetria in upper limbs. She needed a wheel chair for ambulation (EDSS: 7). The patient started treatment with two 5 day cycles of iv steroid pulses; NTZ was restarted 2 months later with progressive improvement (EDSS: 3.5) and no new relapses.

### 2.4 Case report 4

A 32‐year‐old female was diagnosed of MS in 2007 after myelitis. She started treatment with AG but she was switched to NTZ in July 2008 due to persistence of clinical activity (severe relapse with myelitis) and disability progression. In November 2011 NTZ was stopped because of the presence of two risk factors for PML (>2 years and JCV+) she was included in a clinical trial. During the first 4 months, she received placebo, starting FTY in March 2012, no bridging therapy was initiated (EDSS: 3.5). In March, she complained of cognitive worsening and weakness in lower limbs, she was unable to read, presented a severe inattention and behavioral changes (EDSS: 5); a neuropsychological study was conducted, with normal processing speed, verbal fluency and abstract reasoning. A moderate impairment of visuospatial processing information and visual‐constructional execution with dysgraphia was present. Also, Difficulty encoding and retrieval in verbal memory with conservation of such processes in visual memory implying bilateral hemispheric lesion. A MRI was performed showing multiple T2 lesions; 45 lesions presented post contrast‐enhancement (Figure 1g,h), JCV in CSF was negative. The treatment with IV MTP was started with progressive improvement. Five months later the patient was able to walk without aid although behavioral alteration and cognitive decline have not been recovered, EDSS: 4.

## 3 Results

Four patients with a mean disease duration of 9.5 years (*SD*: 4.12) were switched from NTZ to FTY due to PML risk (mean time on NTZ of 3.1 years, all patients anti‐ JCV+, at that time the JCV‐index status was not available). The patients presented a mean of 1.5 relapses prior to NTZ therapy. FTY was started in the 3–4 following months after discontinuation. Despite the treatment, the patients presented neurological deterioration (mean: 3.5 months, *SD*: 2.08) with multifocal involvement: 75% presented motor disturbances, 50% cognitive impairment, and 25% seizures. Mean EDSS worsening was of 3.25 points (*SD*: 2.33). The MRI showed a huge increase in T2 lesions and Gd enhanced lesions (mean: 23.67, *SD*: 18.58; Figure 1). All patients received 5 days of IV MTP, one patient required PLEX. After the rebound all the patients presented neurological deterioration (mean: EDSS 1.125; *SD*: 0.478). Three patients continued treatment with FTY with a positive outcome, only one patient restarted NTZ.

## 4 Discussion

The existence of a rebound phenomenon after NTZ withdrawal is controversial. Several open studies and case reports have reported an increased clinic radiological activity after NTZ suspension beyond pretreatment levels (Beume et al., 2015; Daelman et al., 2012; Gueguen et al., 2014; Jander et al., 2012; Kerbrat et al., 2011; Lenhard et al., 2010; Miravalle et al., 2011; Papeix et al., 2011; Rigau et al., 2012; Rinaldi et al., 2012; Salhofer‐Polanyi et al., 2014; Sorensen et al., 2014; Vellinga et al., 2008; West & Cree, 2010). In a Danish cohort, 83/375 (22.1%) of patients presented a rebound after stopping the treatment (Sorensen et al., 2014). Another analysis of 200 patients who discontinued NTZ, 11.9% present a rebound activity (Salhofer‐Polanyi et al., 2014). Recently, an analysis of 47 NTZ withdrawals found a significant clinical worsening (defined as a two‐step EDSS increase) in 19% of patients (Vidal‐Jordana et al., 2015). Nevertheless, other studies pointed to a restoration of the inflammatory levels to pretreatment levels not exceeding the prior activity. A post hoc analysis from the AFFIRM, SENTINEL, and GLANCE studies showed a return of disease activity independently of receiving alternative treatment or not (O'Connor et al., 2011). The RESTORE study, a randomized 24‐week NTZ treatment interruption study, observed that up to 29% of patients after NTZ discontinuation showed an MRI disease recurrence and 15% had a clinical relapse (Fox et al., 2014). The TY‐STOP, an observational study, revealed that in the first year after NTZ cessation, up to 35% of patients had a relapse (Clerico et al., 2014). None of these studies described a rebound phenomenon.

These differences should come from the different study settings. The studies revised by O'Connor et al. (2011) were prior to the description of PML when NTZ was not restricted to groups of patients with more aggressive disease; Moreover, the RESTORE study, clinical (defined as a relapse the prior year) or radiological (defined as Gadolinium‐enhancing lesions on screening) activity were considered exclusion criteria so only stable patients were analyzed.

The return of disease activity probably reflects an accelerated influx of these immune cells and cytokines into the brain through the endothelial membranes (O'Connor et al., 2011). The mechanism of action of NTZ is not clearly understood; inhibitions of the adhesion to the VCAM at the endothelium with the consequent decreasing in the extravasations to adjacent tissues play an important role. Recently, patients in long‐term treatments with NTZ showed a reduction in CD4 and CD8 lymphocytes, CD19 B‐cells and CD138 plasma cells in the CSF (Stuve et al., 2006) with increased percentage of activated CD4 and CD8 cells expressing proinflammatory cytokines in peripheral blood. This may be due to the diminished extravasation to the CNS (Kivisakk et al., 2009). Also, increased IL‐17, IL‐2, and IL‐1βafter 1 year of treatment have been demonstrated (Oreja‐Guevara et al., 2012; Ramos‐Cejudo et al., 2011). The cessation of the NTZ may lead to an increased permeability of the blood‐brain barrier with the consequent massive transfer of activated lymphocytes form peripheral blood to CNS. Stuve et al. (2006) reported a clinical MS relapse in a patient with the highest total CSF CD4 and CD8 T‐cell count after cessation of NTZ therapy, suggesting that a more rapid return of immune surveillance and function may be associated with IRIS in patients with MS.There are few data about the neuropathological findings; in previous case reports a severe demyelinating immunopathologic pattern I lesions were described (Beume et al., 2015; Daelman et al., 2012; Lenhard et al., 2010) with a dense T‐cell infiltrate dominated by CD8 although cases with CD4 predominance have been described (Rigau et al., 2012) indicating probably a reactivation of the disease. Thus, debate continues among neurologists as to whether some of the clinical worsening in patients after NTZ withdrawal is caused by CNS‐IRIS or worsening of their MS.

The rebound rate varies among studies between 10% and 30% of patients (Gueguen et al., 2014; Havla et al., 2011; Kerbrat et al., 2011; Miravalle et al., 2011; Rinaldi et al., 2012; Salhofer‐Polanyi et al., 2014; Sorensen et al., 2014; Vidal‐Jordana et al., 2015). The reactivation of the disease activity occurs frequently within the 3‐6 months after NTZ cessation, in relation with the drug pharmacodynamics features (Kerbrat et al., 2011; O'Connor et al., 2011). Cree et al. (2013) evaluated biomarkers of NTZ treatment (lymphocyte counts, alpha4‐integrin saturation, sVCAM, and CD49d expression) and demonstrated that 4 months after discontinuation all the biomarkers returned to the same levels as in untreated patients.

Fast reintroduction of alternative treatment after NTZ cessation is recommended to reduce the risk of recurrence. However, there is no consensus on the appropriate management. Some studies have assessed the usefulness of different strategies, nonetheless, a bridging therapy with pulsed IV MTP or switching to disease‐modifying treatment have not proved to be effective enough in controlling the disease (Havla, Keliter, & Kümpfel, 2013). Up to 50% of patients switched to FTY, a second‐line treatment for RRMS, presented a relapse, preferably during the firsts weeks (Cohen et al., 2014; Havla, Keliter, et al., 2013; Havla, Tackenberg, et al., 2013; Rinaldi et al., 2012; Vidal‐Jordana et al., 2015); besides, rebound cases after FTY initiation have been described (Jander et al., 2012; Papeix et al., 2011).

There are controversial data on factors predisposing to recurrence, however some studies suggest that pretreatment activity, longer MS duration (Havla, Tackenberg, et al., 2013; Lenhard et al., 2010; Miravalle et al., 2011), increased activity in the prior months to cessation (Jokubaitis et al., 2014) and longer washout periods are the main predisposing factors. Classically, a 3‐ to 6‐month washout period has been recommended for FTY switching (Fazekas et al., 2013; Jeffery et al., 2011). Nevertheless, recent evidence suggests the need for shorter washout periods. O'Connor et al. (2011) pointed out that NTZ was cleared from peripheral blood by 2 months from the last infusion (O'Connor et al., 2011). The TOFINGO study (Disease control and safety in RRMS patients switching from NTZ to FTY: a 32‐week, rater‐ and patient‐blind, randomized, parallel‐group study) demonstrated that the 8‐week over the 12‐week washout group had least activity over 8 weeks of FTY treatment and over 24 weeks since the last NTZ dose, the lowest change in T2 lesion volume from baseline to week 24 and the highest proportion of patients free from Gd‐enhancing lesions at week 24 (Kappos et al., 2013). Moreover, other series supports that initiating FTY within the first 3 months is associated with a significantly lower risk of relapse (Cohen et al., 2014; Havla et al., 2011; O'Connor et al., 2011). In our series, all the patients started FTY after 3 months, classically recommended as washout period.

There are no randomized controlled trials or established guidelines about the correct treatment in a rebound. The most frequent treatment is pulsed IV MTP for 5 days. The usefulness of PLEX is controversial with some reports of worsening maybe due to a rapid NTZ clearance in peripheral blood and an increased flux of lymphocytes into the brain (Lenhard et al., 2010). Our patients had severe deterioration during the rebound; however, despite a mild improvement after the rebound treatment, significant disabilities (exceeding pre‐NTZ treatment) were maintained over time.

## 5 Conclusion

After stopping NTZ, disease activity may return to pretreatment levels or a severe rebound with a marked clinical and radiological worsening could be seen. Prior to NTZ withdraw, a risk benefit balance should be evaluated and, if cessation is mandatory, a close monitoring and an early initiation of alternative treatment are recommended after drug withdrawal.

## Conflict of Interest

The authors declare no conflict of interest.
